# Enhancing Learner Motivation and Classroom Social Climate: A Mixed Methods Approach

**DOI:** 10.3390/ijerph17155272

**Published:** 2020-07-22

**Authors:** Alfonso Valero-Valenzuela, Oleguer Camerino, David Manzano-Sánchez, Queralt Prat, Marta Castañer

**Affiliations:** 1 Faculty of Sciences of the Sport, University of Murcia, 30720 Murcia, Spain; avalero@um.es (A.V.-V.); david.manzano@um.es (D.M.-S.); 2 National Institute of Physical Education of Catalonia (INEFC), University of Lleida (UdL), 25192 Lleida, Spain; qprat@inefc.es (Q.P.); mcastaner@inefc.es (M.C.); 3 Lleida Institute for Biomedical Research Dr. Pifarré Foundation (IRBLLEIDA), 25198 Lleida, Spain

**Keywords:** teaching strategies, motivational mechanisms, observational analysis

## Abstract

The aim of this study was to analyze how motivation and classroom social climate was enhanced in the teaching–learning context throughout a Pedagogical Model of Personal and Social Responsibility (TPSR) implementation using a mixed method approach. An educational program was applied during an academic year in a student sample of primary and secondary school. A total of 44 sessions with 54 participants, between 11 and 16 years old (*M* = 13.41 years, *SD* = 1.73) were video-recorded. A multilevel triangulation design of mixed method research was applied to merge: (a) the Observational System of Teaching Oriented Responsibility (OSTOR), which revealed how the students’ behavior patterns shifted an alongside the interventions with (b) a set of five complementary questionnaires: Motivation toward Education Scale (EME), Responsibility Questionnaire (PSRQ), Basic Psychological Needs Questionnaire (PNSE), Questionnaire to assess social school climate (CECSCE) and Questionnaire of School Violence (CUVE). The mixed methods design confirmed that both the observational and the inferential analysis show an improvement of the TPSR implementation in the student’s responsibility and satisfaction and the social climate of the classroom. The other variables, although they were also improved, did not do it significantly; all the motivation dimensions showed higher values, except for amotivation and violence.

## 1. Introduction

### 1.1. The Classroom Social Climate

The term *classroom social climate* (CSC) refers to how students and teachers perceive the quality of their experiences in the classroom. How they feel ultimately determines their behavior in this setting [1]. Classroom climates depend on a complex ecological framework that includes self-efficacy and the different socioemotional factors that influence this construct [2]. In addition to improving student motivation [3,4] and basic psychological needs (autonomy, competence, and relatedness) [5], a positive classroom climate stimulates learning and improves academic outcomes [6,7].

López et al. [8] found that CSC was one of the strongest predictors of emotional competence, which is essential for good academic performance. Teachers thus need guidance on how to create a positive socioemotional climate in order to improve academic achievements Garrido et al. [9] detected a clear link between better academic performance and higher learning strategy scores, increased motivation, and a better school climate. In a study of bullying in schools, Polo et al. [10] concluded that the only way to improve the climate in classrooms and prevent bullying was through innovative educational strategies.

### 1.2. Determinants of Classroom Social Climates

CSC depends on sociocultural and pedagogical factors. While sociocultural factors affect academic achievement [11], they are not directly modifiable in the classroom, unlike pedagogical factors, which can be modified, both in the classroom and the larger school setting, through motivation [12], the fostering of autonomy [13] and responsibility [14,15], the satisfaction of basic psychological needs [16], and the reduction in violence [17].

CSC has two dimensions. On the one hand, they involve interactions between individuals, each with their own values, emotions, needs, knowledge, and prior experiences. These interactions generate a dynamic process of social construction that leads to a greater sense of belonging and improved communication [18], in addition to better conflict resolution skills and a greater perception of institutions as organizations [19]. On the other hand, they have a dimension that can be negatively affected by a number of factors, such as indiscipline, impulsiveness [20], violence, and conflict [19].

### 1.3. Classroom Social Climates and the Teaching Personal and Social Responsibility Model

The Teaching Personal and Social Responsibility (TPSR) model improves prosocial behavior and classroom climates [21,22], and is one of the most powerful tools for promoting self-autonomy in students [23].

The model has five levels, which are well described in the literature [24]. The idea is that students gradually work their way up through the levels, but within a flexible set-up that allows them to return to a previous level when necessary. Students at level 0, *irresponsibility*, for example, do not take responsibility for their acts and blame others. When they reach level 1, *respect for the rights and feelings of others*, they use negotiation and dialogue to resolve conflicts and disagreements and show respect for other people’s qualities and characteristics. At level 2, *participation and effort*, they are willing to make an effort to achieve goals and they show interest in activities, regardless of whether or not these are aligned with their preferences. At level 3, *self-direction*, they show autonomy and take ownership of their learning, without instruction from their teachers, while at level 4, *caring and leadership*, they show empathy and commitment to others without expecting anything in return. Finally, at level 5, *transfer*, they are capable of transferring what they have learned at previous levels to contexts outside the classroom (e.g., friends, family, and formal and non-formal educational activities).

The aim of this study was to determine the effects of a primary and secondary school intervention aimed at increasing learner autonomy and personal and social responsibility on classroom social climate and its determinants, motivation, basic psychological needs (autonomy, competence, and relatedness), and levels of violence.

## 2. Materials and Methods

### 2.1. Research Design

**Figure 1:**
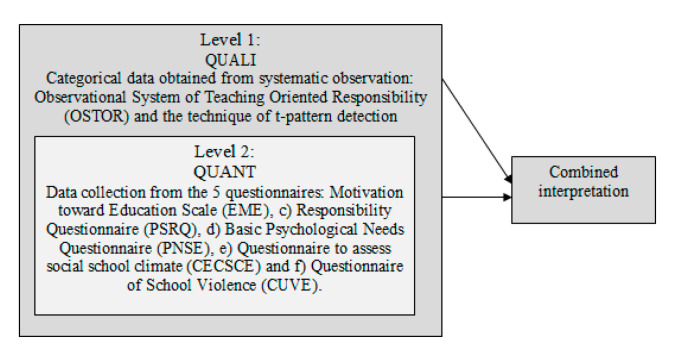
Multilevel triangulation design adapted from Creswell et al. [25] (p. 64) and Camerino et al. [26] (p. 12).

The use of mixed methods approaches allows for integrating qualitative and quantitative data, and they are increasing exponentially [25] in several disciplines. Education is not an exemption, and those that have used the TPSR model have begun to apply mixed methods approaches that incorporate observational methodology [26], demonstrating them to be suitable to obtain a broader analysis of the data (i.e., [20,21,22,23,24,25,26,27,28,29]) and proving them to be effective in previous related research such as teacher’s communication [18,30] and TPSR pedagogical research [23,31,32]. The mixed methods approach incorporates different types of designs [25,26]. In this study, we conducted a multilevel triangulation design (see Figure 1) in which the main tool is the systematic observation conducted alongside the educational program and the 5 questionnaires administered to the students at the beginning and at the end of the program to detect the determinants of CSC.

### 2.2. Participants

An educational program was applied in a primary and a secondary school in a total of 44 sessions—11 for each teacher—during an academic year. Participants were students and their teachers and they both were selected by accessibility and convenience.

The teachers: a total of four teachers, with a level of experience between 5 and 10 years of teaching in their subjects, were video-recorded and analyzed in every 44 sessions (11 sessions per teacher with a duration of 55 min). The selection criteria for them was to choose those interested in following a continuous professional development during the treatment. Two teachers taught Physical Education (PE), one taught History and taught Language. One of the PE teachers was an experienced teacher in TPSR; meanwhile, the rest were inexperienced teachers in TPSR.

The students: the group was composed of 54 students, between 11 and 16 years old (*M* = 13.41 years, *SD* = 1.73) One class out of all the classes that each teacher had was randomly selected. For student age selection, as a point of interest we included the first stage of secondary education, defined according to current legislation in Spain [33], along with the final year of primary education, which would mark the boundary between the penultimate and final stages of Piaget’s cognitive development [34].

Both the informed parental consent related to the students and the signed consent form from teachers were obtained in writing. Furthermore, they were informed in accordance with the Declaration of Helsinki, and this study was accepted and verified by the Ethics Committee of the University of Murcia, Spain (ID 1685/2017).

### 2.3. Instruments and Measures

To conduct the mixed methods design explained above, we used six instruments: (a) the Observational System of Teaching Oriented Responsibility (OSTOR) [23] adapted from the Spanish version, SORPS [32], was used to obtain teacher behavior patterns alongside all the sessions of the program and the questionnaires, (b) Motivation toward Education Scale (EME), (c) Responsibility Questionnaire (PSRQ), (d) Basic Psychological Needs Questionnaire (PNSE), (e) Questionnaire to assess social school climate (CECSCE) and (f) Questionnaire of School Violence (CUVE) were administered at the beginning and at the end of the program.

#### 2.3.1. Observational System of Teaching Oriented Responsibility

The OSTOR [23] (Table 1) is comprised of six criteria. The first four criteria are related to teacher actions: (1) Expectations; (2) Explanations; (3) Organization; and (4) Task adjustments. The fifth criterion is related to the student: (5) Student’s responses. The last criterion is related to how the session concludes: (6) Session summary) each criterion was expanded to build an exhaustive and mutually exclusive observation total of 18 categories.

#### 2.3.2. Recording Instrument Software LINCE PLUS

The teaching behavior sequences, session by session, were coded using the free instrument software LINCE PLUS [35]. This software was designed to facilitate the systematic observation of spontaneous behaviors in any situation or habitual context. It is highly practical and easy to use and integrates a wide range of functions: coding, recording and enabling data export to several data analysis applications. In addition, LINCE PLUS [35] allows us to obtain a data quality check between observers. The data obtained were automatically exported to the THEME software package [36] for T-pattern detection.

#### 2.3.3. Questionnaires

We administered a set of five complementary questionnaires:

(1) Personal and Social Responsibility Questionnaire (PSRQ): to measure personal and social responsibility. It was adapted to the school context by Li et al. [37] and into Spanish by Escartí et al. [38], with a Likert-type scale with a minimum value of 1 and a maximum of 5 and a total of 14 items, with seven to evaluate each responsibility: personal responsibility (e.g., “I respect others”) and social responsibility (e.g., “I effort”). All the questions came under the premise of “In my Classes...”, an example of the item would be “Respect for others”. Reliability in the pre-test and post-test was 0.76 and 0.87 for social responsibility and 0.83 and 0.86 for personal responsibility, respectively.
(2) Psychological Need Satisfaction in Exercise (PNSE): to measure the satisfaction of the need for social competence, autonomy and relatedness. The scale was adapted for Spanish and to the education context by Moreno et al. [39,40] and validated in a 12–16 years old sample. This scale consists of 18 items, six to evaluate each need: competence (e.g., “I am confident to perform the most challenging exercises”), autonomy (e.g., “I believe I can make decisions during the training sessions”) and relatedness (e.g., “I feel attached to my training mates because they accept me as I am”). All the questions came under the premise of “During my class” and the answers were provided on a Likert-type scale ranging from 1 (False) to 6 (True). Reliability in the pre-test and post-test was 0.76 and 0.84 for autonomy, 0.67 and 0.74 for competence and 0.73 and 0.80 for relationships, respectively. Moreover, the psychological mediator index (PMI) was applied to evaluate the three variables together, with a consistency of 0.83 and 0.89 in the pre and post-test, respectively.
(3) Motivation toward Education Scale (in French, EME): to measure motivation. To measure the student’s academic motivation, the Échelle de Motivation en Éducation [41] was used with the translated and validated version of Núñez et al. [42]. The instrument is made up of 28 items, preceded by the phrase “I go to school/institute because…” with a Likert-type scale of five points, from 1 (it does not correspond at all) to 5 (it corresponds totally) and distributed in seven subscales. The internal consistency analysis yielded the following values in the pre-test and post-test, respectively: 0.85 and 0.90 for intrinsic motivation to know (e.g., “because my studies allow me to continue learning a lot of things that interest me”), 0.78 and 0.77 to experience sensations (e.g., “for the pleasure of reading interesting authors”), 0.85 and 0.87 to accomplish (e.g., “for the satisfaction I feel when I improve in my studies”), 0.92 and 0.94 for general intrinsic motivation (means of intrinsic motivation to know, to experience and to accomplish), 0.77 and 0.77 for identified regulation (e.g., “because it will help me to choose better my professional orientation”), 0.75 and 0.69 for external regulation (e.g., “to have a better salary in the future”), 0.74 and 0.84 for introjected regulation (e.g., “because I want to prove myself I am capable of succeeding in my studies”) and 0.79 and 0.84 for amotivation (e.g., “at that time, I had good reason to go to high school; but, now I wonder if I should continue in it”). On the other hand, the self-determination index (SDI) was applied using the formula ((intrinsic motivation × 2 + identified regulation) + (introjected regulation + external regulation)/2 − (amotivation × 2)), it had an internal consistency of 0.85 and 0.87 in the pre-test and in the post-test, respectively.
(4) Questionnaire of School Violence (Cuestionario de Violencia Escolar, CUVE): to evaluate violence perception. The questionnaire was made by Álvarez et al. [43] and was adapted to Spanish and to primary and secondary education contexts by Álvarez et al. [44]. This questionnaire has two versions (for primary and secondary school) and we used both depending on the sample. Answers are provided with a Likert-type scale from 1 (totally disagree) to 5 (totally agree). On the other hand, the internal consistency of the pre-test and post-test for primary school was 0.96 and 0.98 and 0.84 and 0.98 for secondary school, respectively.
(5) Questionnaire to assess social school climate (CECSCE): to evaluate the climate perceived by the students. It was prepared by Trianes et al. [45]. The questionnaire has two subscales called “Climate relative to the school” (e.g., “when there is an emergency, there is someone to help me”) and “Climate relative to the teaching staff” (e.g., “when students break the rules they are treated fairly”) with eight and six items, respectively. The scale uses a five-point Likert scale from 1 (totally disagree) to 5 (totally agree) The internal consistency was determined in the pre-test and post-test with values of 0.83 and 0.87 for school climate and 0.73 and 0.80 for teacher climate, respectively. The values for general classroom climate were 0.85 in the pre-test and 0.91 in the post-test.

### 2.4. Procedure

#### Recording Procedure

To familiarize students and avoid reactivity behaviors versus being observed, a camera for video recording was installed in the classroom during several sessions previous to the beginning of the educational program. The total of the 44 sessions—11 for each teacher—were registered from the moment the teacher effectively started the educational activities of the session. Two experts, with a PhD on physical education, coded the categories of OSTOR system by means. LINCE PLUS was also used to obtain the reliability between the observers and the fun, resulting in a kappa statistic of 0.95 for inter-observer and 1.98 for intra-observer analysis.

On the other hand, the questionnaires were administered to the students under the supervision of some of the members of the investigative team together with the presence of one of the teachers who implemented the TPSR. The teacher assured the anonymity and sincerity of the answers. Meanwhile, the investigator was attentive to be able to settle those doubts that could be generated during the process and how to create a relaxed and distended ambience to enhance the concentration and to assure the maximum veracity in the answers.

## 3. Data Analysis

Data analysis was conducted applying the T-pattern detection technique and descriptive and inferential analysis from the results of all questionnaires.

### 3.1. T-Pattern Detection

The observational data obtained from LINCE PLUS were directly exported to THEME software package [36] for T-pattern detection. T-pattern detection (temporal pattern detection) [35,46]) is a relevant data analysis technique in systematic observation. T-pattern analysis detects the temporal structure of complex behavioral observable sequences. THEME software is a powerful research tool for obtaining T-patterns that allows exploring accurately the stronger connections between successively recorded behaviors and underlying pedagogical actions, as related studies have conducted [18,20,23,29,30,32,47,48]. THEME software uses an algorithm to compare all coded behaviors from the simplest to the most complex combinations throughout the detection of a statistically significant chain of behaviors from a great amount of behavioral events. This software randomizes and re-analyzes the original data repeatedly using the same search parameters [46] to reinsure T-patterns are not discovered only by chance. In addition, as Magnusson stated [36], three criteria were applied to guarantee this: (a) presence of a given T-pattern in at least 25% of all sequences, (b) a significance level of 0.005, and (c) a redundancy reduction setting of 90% for occurrences of similar T-patterns.

### 3.2. Analysis from Questionnaire

First, the normality hypothesis was tested with the Kolmogorov–Smirnov test (*p* < 0.05). In terms of nonparametric tests, the Wilcoxon signed-rank test was used to compare pre- and post-test variables. The degree of change in every group was obtained through the size effect using Cohen’s formula [49]. Values of 0.2 and 0.5 were considered as a small effect, between 0.5 and 0.8 as medium, and more than 0.8 as large. Furthermore, frequency and percentage analyses were conducted to evaluate the teacher’s behavior, as well as a description of the interviews through qualitative analysis with the aim to get an internal perspective of the experience at the end of the process [50]. The softwares IBM SPSS 22.0 (SPSS Inc. Chicago, IL, USA) and Excel 2010 (Microsoft, Redmond, DC, USA) were used to analyze the data from the questionnaires and the observation instrument, respectively.

## 4. Results

### 4.1. Evolution Behavior from T-Patterns Detected

**Figure 2:**
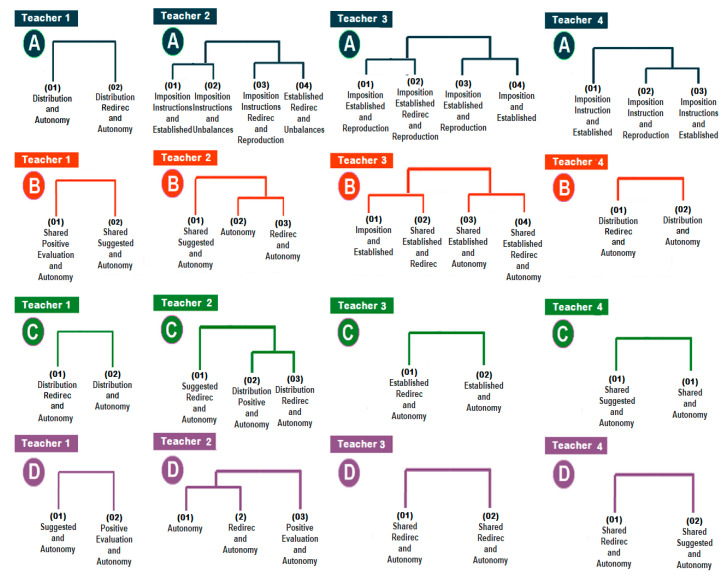
The T-patterns of the four teachers (1, 2, 3 and 4) throughout the four levels (A, B, C and D) of TPSR implementation.

The implementation of the TPSR of the four teachers caused an evolution of the levels of responsibility of the students. In Figure 2, we represent the T-patterns that show this progress; in the first level (A) of the TPRS, the answer of the students is still based on reproduction tasks before an imposition or instruction task; in the second level (B) and the third (C), the behaviors show the corrections and suggestions of the teacher, more autonomous students and of major participation and effort; in the fourth level (D), teachers use positive evaluations and shared proposals, enhancing autonomy in all the cases.

### 4.2. Results from Questionnaire.

Pre- and post-test means and standard deviations of all the variables are shown in Table 2, which also includes the *p*-values obtained with the nonparametric comparative test and the effect size with Cohen’s formula.

### 4.3. A Comparison of the Pre-Test Variables

The statistically significant differences were observed in autonomy (*p*-value = 0.000), competence (*p*-value = 0.007), psychological mediator index (*p*-value = 0.000), teacher climate (*p*-value = 0.020), school climate (*p*-value = 0.001), classroom climate (*p*-value = 0.001), social responsibility (*p*-value = 0.007). Furthermore, although not reaching 5% significance, personal responsibility showed a *p*-value which was close to that level (*p*-value = 0.050), and the effect size was medium.

### 4.4. Discussion

The main objective of this study was to verify how the application of an educational program based on increasing the responsibility levels through the TPSR improved the CSC, as well as the factors that determine it; motivation, basic psychological needs, and education of violence levels. The findings obtained fit with different studies that revealed the advantages that can be achieved with TPSR in an educational context related to the improvement of the classroom climate and personal and social positive values such as responsibility, autonomy or competence [23,51,52].

Despite the fact that there are still few former studies that have implemented the TPSR in different subjects, they reported improvements in some of the student motivation dimensions, specifically, improvements in intrinsic and introjected motivation [53] or in the self-determination index and reduction in the amotivation values [54]. However, in the present work, no significant changes over time were achieved in students who received this program, also reporting a low effect size in all its dimensions; therefore, it is not attributable to the sample size either.

On the other hand, no improvements were found in the violence perception, despite the fact that former studies in the PE field, such as [55,56,57,58], related higher responsibility and sportsmanship levels with lower violence and disruptive behavior levels. In line with the present work, similar results have been already obtained without changes in the violence perception in the study by Manzano-Sánchez and Valero-Valenzuela [54], where, despite the decrease in antisocial behavior, there was no change in the student violence perception who received the TPSR program. It should be noted that the same results were also reported in studies focused only on the PE field, where it was found that personal and social responsibility is capable of negatively predicting attitudes of violence, but where the intervention study that was associated with it did not find decreases in this variable [21].

In some studies, the lack of improvements in the motivation variable that have been reported could be due to the short time of the intervention. There are several studies which alluded to the lack of time during the implementation for the program as the main reason why no significant changes are achieved in the variables studies [54,59,60], despite the fact that for teachers who implement this new program, it is motivating [61]. However, in this research, the intervention program lasted a whole school year, so other factors such as the kind of motivation measured (PE motivation versus academic motivation), instruments used (questionnaire to assess motivation in the PE class or perceived locus of causality versus EME) [62,63], or the sample size could be behind these results [53].

With regard to the basic psychological needs satisfaction, improvements were obtained in autonomy and competence, as well as in the psychological mediators’ index. These results are positive and are in line with those found by Manzano and Valero-Valenzuela [53], who achieved improvements in the only basic psychological need measured, which was autonomy, and with Manzano-Sánchez and Valero-Valenzuela [54], who found improvements in the psychological mediators index for both primary and secondary school students. Some research in the PE field, when they applied the TPSR, obtained higher values in all, [32] or at least in some of them [57].

Improvements were achieved in personal and social responsibility (although the latter was at the limit of significance); these results are in line with many works which already contributed to the school environment in PE field [51,64,65], in addition to recent work covering other subjects apart from PE, such as those of Manzano and Valero-Valenzuela [53] and Manzano-Sánchez and Valero-Valenzuela [54], with increases in social and/or social responsibility in relation to groups that did not receive this kind of methodology. In this regard, authors such as Escartí et al. [52], who were pioneers implementing the program in different curricular subjects in primary school in addition to PE, warned of the difficulty teachers had in being faithful to and using certain strategies (especially the transfer of responsibility levels to other contexts), and therefore warned of the need for continuous development programs that ensure adequate teacher training for implementing TPSR in the classroom.

As for the CSC, there was a significant improvement in all its dimensions (school climate, teacher and classroom climate), which is very interesting to take into account because of the repercussions it has on learning and academic performance [6,7,8]. The data obtained here coincide with the results of Manzano and Valero-Valenzuela [53] and Manzano-Sánchez and Valero-Valenzuela [54], who obtained improvements for these variables in primary and secondary students. Furthermore, following these studies, it is worth noting that those groups who did not receive this TPSR-based methodology, did not report changes and if there were any, they were to worsen with respect to the starting levels. Manzano-Sánchez et al. [58], in a study aimed at finding out the opinions of teachers who had been applying the program for eight months in different subjects of the secondary schools curriculum, reported a very positive assessment of the coexistence improvement next to a model satisfaction. In line with this, Hortigüela et al. [66], in a study with PE teachers from different countries, found positive change in their perception of social goals and perspectives on discipline strategies. Therefore, specific pedagogical programs for the CSC are necessary if it is to be achieved in a practical way, making the TPSR an effective program for its achievement. In sum, different studies have shown that TPSR teaching intervention, due to the motivational strategies of its different levels, produces an increase in student participation and involvement towards autonomy [28,67]. Our mixed methods interpretation of the results verified this evolution towards autonomy, which consequently favors the improvement of CSC and all its factors.

One of the limitations of the present study that could mean that no more significant differences were found in some of the variables is the participants’ educational level, since students of two distinct stages (primary and secondary schools) were analyzed together. Small differences of age could suppose great changes in their evolutionary development. In future investigations, it is highly recommended that the research focus on a more specific age group, or perform analyses differentiating according to age or at least educational stage. In addition, it would also be very interesting to analyze the results according to gender, since former studies have shown differences in some of these variables depending on whether the students were males or females [60]. Moreover, this method of analysis could be related with studies that take into account emotional or behavioral problems in such a particular stage of adolescence [68].

Finally, assuming that the selection of the sample was done for accessibility and convenience, an unavoidable condition on most occasions when working with natural groups of students in schools, a proposal for improvement in new research is to have a control group that allows for the comparison of the results obtained by students when have received the TPSR with those who have continued to receive the teaching used at that moment. It will be particularly interesting to identify the methodological strategies applied by teachers in the classroom according to the methodology they are using, and to observe possible differences in student behavior patterns.

## 5. Conclusions

The mixed methods approach used to analyze changes over time in a group of primary and secondary students was effective in identifying improvements in personal and social responsibility, in the students’ autonomy level and in the classroom social climate. Furthermore, these changes were accompanied by an increase in the satisfaction of several basic psychological needs, namely, competence and autonomy. Therefore, the TPSR is outlined as a school project suitable for use by teachers not only in PE, but also of other subjects, and can be considered as a global strategy to improve the climate of coexistence in an educational centre. Nowadays, this will also mean a change in educational and pedagogical policies related to the approaches and models of the educational systems towards a demand for greater participation and responsibility of the students in an increasingly virtual educational intervention.

Other expected changes in the variables of motivation and perception of violence did not occur, despite an improvement in their scores, which could be due to the use of different measurement tools than those used in previous studies or to a reduced sample size. Therefore, for future studies, it is suggested to increase the number of participants, since significant differences in the statistical tests used depend in part on the sample size. Furthermore, we must be cautious in the statements made due to the absence of a control group that prevented us from contrasting these results with the treatment received and ruling out that other kind of contaminating variables could have had an influence.

## Figures and Tables

**Table 1:** Observational System of Teaching Oriented Responsibility (OSTOR) (Camerino et al. [23]).

Criterion	Category	Code	Description
Expectations	Objective of Session	OBS	Prospects and aims of the session
	Objective of Task	OBT	Prospects and aims of the task
Explanations	Imposition Instructions	IMP	Without the possibility to include changes
	Shared	SHA	Proposals are allowed to be decided in common
Organization	Established	EST	Spaces and materials are mandated
	Distribution of Function	DIS	Functions and roles are allocated
	Suggested	SUG	Teachers pose opportunities to pupil’s interventions
Task adjustments	Negative Evaluation	NEG	A rebuke to the students
	Redirect	RED	Correct student’s responses
	Positive Evaluation	POS	Encourage and motivate the students
	Proposals	PRO	Formulate new options to be successful
Student’s responses	Reproduction	REP	Replicate tasks or situations
	Unbalances	UNB	Disarranged or disordered responses
	Autonomy and Leadership	AUT	Drive initiatives
	Self-Assessment	SAS	The student evaluates its own performance
Session summary	Guided Summary	GUS	The teacher summarizes the session
	Shared Summary	SHU	The students take part in the sessions summary
	Nonexistent Summary	NSU	The sessions end without be summarized

**Table 2:** Intervention results.

Variables	Pre-Test		Post-Test		Pre-Post Difference	
	Mean	SD	Mean	SD	*p*-Value	Cohen’s D
IM to know	5.564	1.432	5.866	1.273	0.164	−0.222
IM to experience	4.801	1.491	5.907	1.402	0.386	−0.205
IM to accomplish	5.324	1.447	5.671	1.276	0.116	−0.254
General IM	5.230	1.330	5.545	1.227	0.220	−0.246
Identified R	5.583	1.085	5.857	1.114	0.155	−0.249
Introjected R	5.455	1.237	5.762	1.240	0.196	−0.248
External R	5.912	1.219	6.187	0.884	0.209	−0.258
Amotivation	1.667	1.136	1.536	0.842	0.610	0.130
SDI	7.027	3.784	7.899	3.720	0.130	−0.233
Autonomy	3.436	0.895	4.005	0.9105	0.000 **	−0.629
Competence	3.889	0.715	4.574	0.701	0.007 **	−0.379
Relatedness	4.093	0.846	4.329	0.690	0.092	−0.306
PMI	3.806	0.654	4.164	0.660	0.000 **	−0.545
Teacher Climate	3.907	0.707	4.105	0.716	0.020 *	−0.278
School Climate	3.590	0.804	3.918	0.793	0.001 **	−0.414
Classroom Climate	3.749	0.641	4.012	0.705	0.001 **	−0.390
Social Responsibility	5.650	0.677	5.405	0.652	0.007 **	−0.361
Personal Responsibility	5.040	0.878	5.336	0.700	0.050	−0.373
Violence	2.419	0.787	2.273	0.959	0.183	0.166

Legend: * *p* = < 0.05; ** *p* = < 0.01; SD = standard deviation; SDI = self-determination index; PMI = psychological mediator index.
